# Arch translocation and the intra-arch elephant-trunk technique with collared graft for extended chronic dissecting aortic aneurysm

**DOI:** 10.1186/1749-8090-8-23

**Published:** 2013-01-31

**Authors:** Shigeru Ikenaga, Akihito Mikamo, Tomoaki Kudo, Hiroshi Kurazumi, Ryo Suzuki, Kimikazu Hamano

**Affiliations:** 1 Department of Surgery and Clinical Science, Yamagchi University School of Medicine, Minami-Kogushi1-1-1, Ube, Yamaguchi 755-8505, Japan

**Keywords:** Arch translocation, Collared graft, Extended aortic aneurysm

## Abstract

Management of extensive, chronic, dissecting aortic aneurysms after prior repair of the ascending aorta presents a technical challenge for surgeons. A symptomatic 64-year-old patient was admitted for elective surgical repair of an aortic annular dilatation, causing severe aortic regurgitation, and a Crawford type II extended thoracoabdominal aneurysm, 4 years after he underwent primary repair of an acute aortic dissection. The aorta was diffusely dilated, and there were no sites beyond the distal aortic arch where anastomosis could be performed. We successfully performed total aortic replacement with a 2-stage strategy, using an arch translocation technique and an intra-arch elephant-trunk technique.

## Background

Dissecting aortic aneurysms may be chronic and extensive following prior repair of the ascending aorta; their management may present a technical challenge. The anatomy and orientation of the aorta make it extremely difficult to expose entirely through a single-incision-approach. We successfully performed total aortic replacement with a 2-stage strategy, using an arch translocation technique and an intra-arch elephant-trunk technique.

## Case presentation

**Figure 1:**
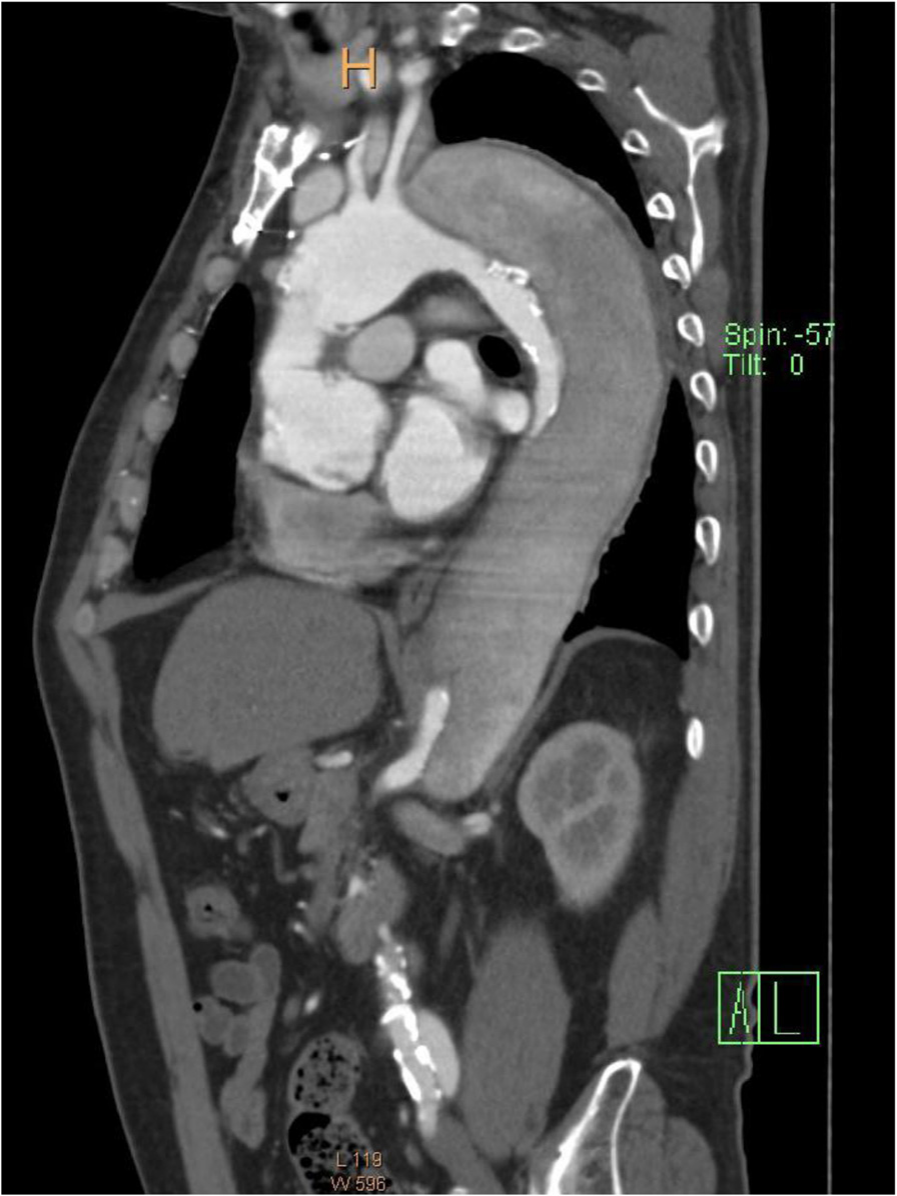
Computed tomography (CT) image showing an aneurysm of the sinus of Valsalva and an extended aortic aneurysm, spanning the proximal descending aorta to level of the abdominal aorta.

**Figure 2:**
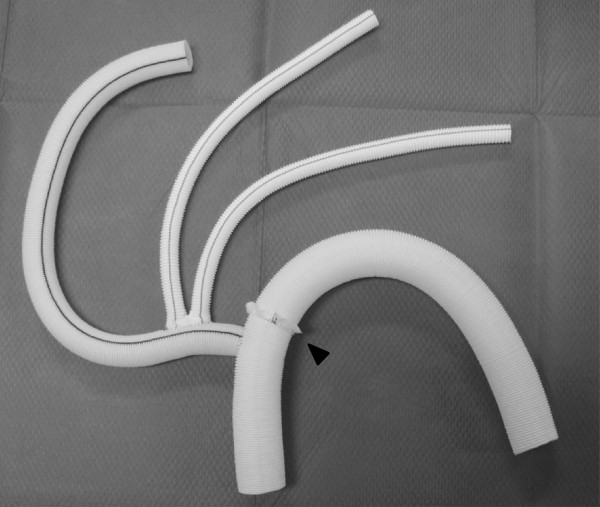
**Collared graft for aortic arch debranching. **The distance from the collar to the distal edge of the graft is 10 cm in length; this part of the graft is inserted into the aorta as a long elephant trunk. Arrow head indicates the collar of the graft.

**Figure 3:**
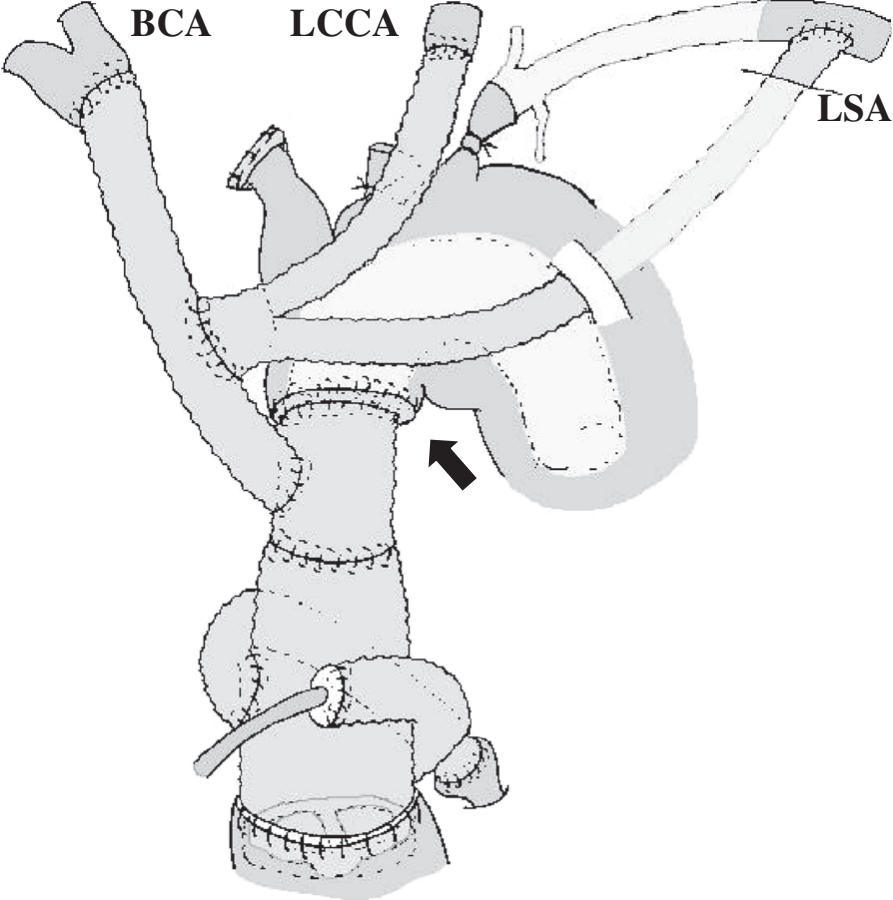
**Schematic of the first operation. **Distal anastomosis is accomplished between the polyester flange and the previous graft. Arrow indicates the distal anastomosis site. BCA: brachiocephalic artery; LCCA: left common carotid artery; LSA: left subclavian artery.

A 64-year-old man had undergone ascending aortic replacement, suspension of the aortic valve, and aorto-coronary bypass to the right coronary artery for acute aortic dissection. Four years later, he presented with shortness of breath and enlargement of the residual dissected thoracic aorta. Computed tomography (CT) revealed an aneurysm of the sinus of Valsalva and an extensive aortic aneurysm, reaching 67 mm in diameter, spanning the length of the descending aorta to the level of the abdominal aorta (Figure 1). Transthoracic echocardiography revealed severe aortic regurgitation. The aorta was chronically, diffusely dilated with no sites beyond the distal arch appropriate for anastomosis; we planned a 2-stage strategy for total aortic replacement. The first surgery began with a median re-sternotomy, followed by exposure of the bilateral axillary arteries, the right common femoral artery, and the right femoral vein. Extracorporeal circulation was established; atrio caval and femoro caval cannulation, in a Y configuration, was used for venous drainage while trifurcated direct bilateral axillary artery and right femoral artery cannulation was used for arterial inflow. Systemic cooling was initiated, with a target rectal temperature of 24°C. The previous graft was clamped and transected above the sinotubular junction, and cold blood cardioplegia was administered in an antegrade fashion to achieve cardiac arrest. The aortic valve was excised, and the aortic root was reconstructed by the modified Bentall procedure using a composite graft (23AHPJ-505 mechanical heart valve; ST. Jude Medical Inc., MN, USA; 26 mm graft; Gelweave, Vascutek Ltd., Inchinnan, Scotland, UK). The coronary arteries were reattached with a polyester interposition graft (10 mm; Gelweave, Vascutek Ltd.). When the proximal anastomosis was completed and the target temperature was reached, systemic perfusion was discontinued. The Brachiocephalic artery and the left subclavian artery were clamped, and 13-French cannulae was inserted into the left common carotid artery. Perfusion of all three head vessel was achieved during selective cerebral perfusion. For the distal anastomosis, we developed an aortic arch translocation technique with a collared graft. Polyester fabric (0.61 mm; Sauvage Filamentous Fabric, Bard Peripheral Vascular Inc., Tempe, AZ, USA) was trimmed in a doughnut fashion to a width of 5 mm. This patch was then attached, similar to a flange, to a 20-mm straight graft (Gelweave, Vascutek Ltd.); this served as the main body of the composite graft. Another 14-mm graft (Gelweave, Vascutek Ltd.), acting as the branch graft, was anastomosed to the main-body graft in an end-to-side fashion for eventual connection with the brachiocephalic artery. Another 16 × 8-mm graft (Gelweave, Vascutek Ltd.) was anastomosed to the branch graft for eventual connection with the left common carotid artery and the left axillary artery (Figure 2). The length of the graft from the flange to the distal edge was 10 cm; this was inserted into the thoracic aorta as a long elephant trunk, and anastomosis was accomplished between the polyester flange and the previous graft (Figure 3).

Four months later, a second operation was performed. With a spinal drainage catheter placed 1 day prior the aorta was accessed through a thoracoabdominal incision with the patient in the right semi-lateral decubitus position. Extracorporeal circulation was established with arterial return cannulae in the right femoral artery and the right axillary artery. Venous drainage was accomplished with 2 cannulae from the right femoral vein advancing into the right atrium and the main pulmonary artery. When the patient’s nasopharyngeal temperature reached 20°C, the descending thoracic aorta was clamped under distal aortic perfusion. After blood circulation was stopped, the thoracic aorta was opened without a proximal clamp and the elephant trunk portion of the graft was grasped and clamped. The thoracoabdominal aorta was reconstructed using a Coselli thoracoabdominal graft (Vascutek Ltd.). The proximal anastomosis was accomplished under antegrade cerebral perfusion from the right axillary artery. The distal anastomosis was accomplished at the level of the abdominal aorta. Three pairs of intercostal arteries were reconstructed, and the visceral vessels were perfused with cold blood before they were individually attached to the main graft.

**Figure 4:**
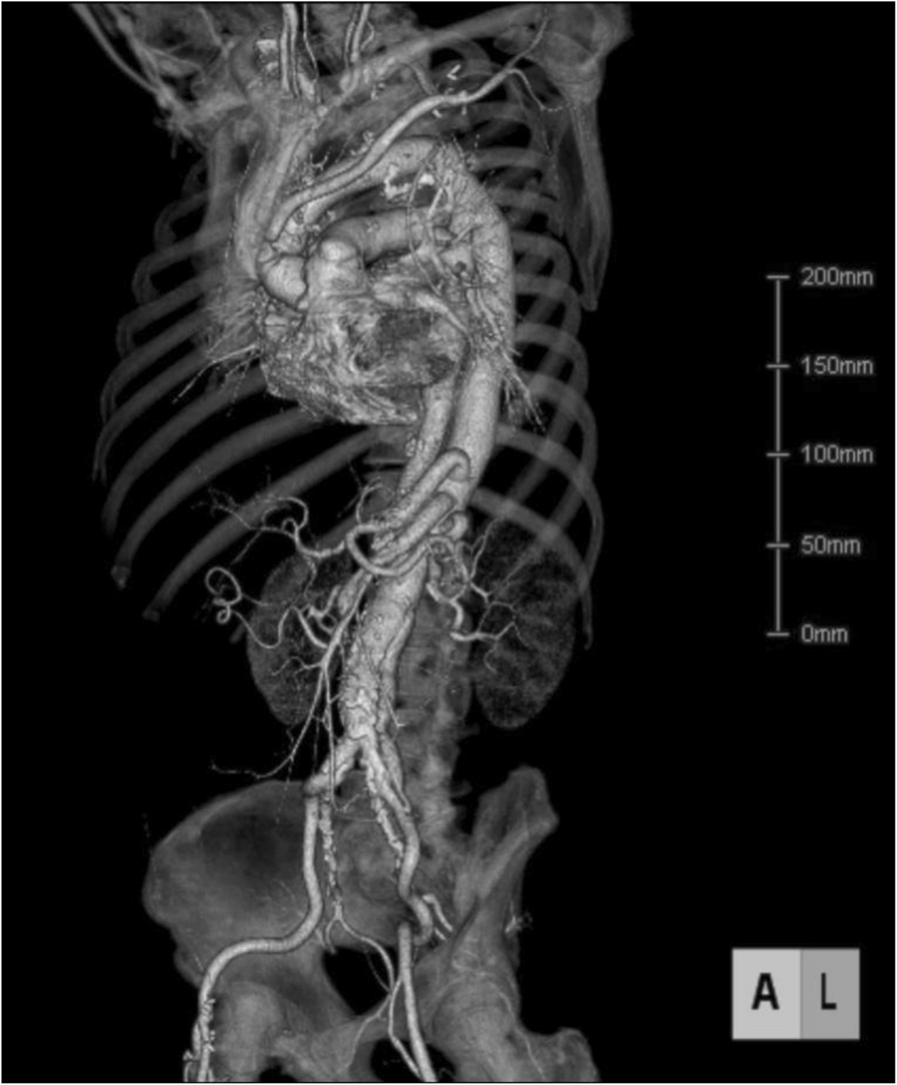
Postoperative CT indicating successful aortic reconstruction.

The patient’s postoperative course was uneventful; postoperative CT indicated successful aortic reconstruction (Figure 4). The patient was discharged on post operative day 19 and is currently asymptomatic, 3 years after surgery.

## Discussion

Although surgical results for acute type A aortic dissections have improved, survivors remain at risk for rupture of residual aortic dissection, requiring further surgical intervention. The incidence of late reoperation after successful surgical treatment is 11- 23% [1-3]. Proximal and distal late reoperations are associated with high mortality rates of 18% and 30%, respectively [1].

Our patient had an aortic annular dilatation, causing severe aortic regurgitation, and a Crawford type II extended thoracoabdominal aneurysm, 4 years after primary repair of an acute aortic dissection. Distal anastomosis was deemed difficult using the normal elephant-trunk method, as there were no appropriate sites beyond the distal aortic arch. We planned a 2-stage strategy for total aortic replacement, with the first stage involving a newly developed arch translocation technique using a collared, long elephant trunk.

Taniguchi et al. first described the arch translocation technique, which avoids contact with the fragile dissected aorta, for total arch replacement [4]. An advantage of their method is that all the anastomoses are visible to the surgeons; any bleeding points can be easily controlled. Although the original technique uses 2 pieces of graft, a 4-branched proximal graft and a long elephant trunk, we simplified this technique by adding a collar for distal anastomosis to a 3-branched graft. This modification reduced bleeding at the distal anastomosis and simplified the second operation.

Safi et al. suggested the usefulness of a 2-stage strategy using the elephant-trunk method for extended thoracic aneurysms, and reported a satisfactory mortality rate [5]. This procedure, however, is only useful in cases with appropriate anastomosis sites in the distal aortic arch. In patients with no appropriate sites for clamping or anastomosis, special efforts are required.

## Conclusion

We have successfully performed total aortic replacement with a 2-stage strategy, using arch translocation and an intra-arch elephant-trunk technique. This technique avoids anastomosis with the fragile portion of the aorta involved with chronic dissection.

## Consent

Written informed consent was obtained from the patient for publication of this Case report and any accompanying images. A copy of the written consent is available for review by the Editor-in-chief of this journal.

## Abbreviation

CT: Computed tomography.

## Competing interests

The authors declare that they have no competing interests.

## Authors’ contributions

SI is a member of medical team and drafted the manuscript. AM is a member of medical team and helped to draft the manuscript. TK is a member of medical team. HK is a member of medical team. RS is a member of medical team. KH is a member of medical team and revised the manuscript. All authors read and approved the final manuscript.
